# National burden of influenza-associated hospitalizations in Cambodia, 2015 and 2016

**DOI:** 10.5365/wpsar.2018.9.5.011

**Published:** 2018-10-23

**Authors:** Vanra Ieng, M Ximena Tolosa, Bunchhoeng Tek, Borann Sar, Kheng Sim, Heng Seng, Miliya Thyl, Chan Dara, Mey Moniborin, Rebekah J. Stewart, Leila C. Bell, Georgios Theocharopoulos, Savuth Chin, Darapheak Chau, A. Danielle Iuliano, Ann Moen, Reiko Tsuyuoka, Erica L. Dueger, Sheena G. Sullivan, Sovann Ly, Disclaimer, The findings and conclusions in this report are those of the authors and do not necessarily represent the official position of the Centers for Disease Control, Prevention

**Affiliations:** a World Health Organization, Country Office, Phnom Penh, Cambodia.; b National Centre for Epidemiology and Population Health, The Australian National University, Australia.; c WHO Collaborating Centre for Reference and Research on Influenza, The Peter Doherty Institute for Infection and Immunity, Australia.; d Communicable Disease Control Department, Ministry of Health, Phnom Penh, Cambodia.; e Centers for Disease Control and Prevention, Country Office, Phnom Penh, Cambodia.; f Angkor Hospital for Children, Siem Reap, Cambodia.; g Svay Rieng Provincial Hospital, Cambodia.; h Kampong Cham Provincial Hospital, Cambodia.; i Centers for Disease Control and Prevention, Atlanta, USA.; j Emerging Disease Surveillance and Response, World Health Organization Regional Office for the Western Pacific, Manila, Philippines.; k National Public Health Laboratory, National Institute of Public Health, Phnom Penh, Cambodia.; l Department of Epidemiology, University of California, Los Angeles, USA.; m School of Population and Global Health, University of Melbourne, Australia.; * Joint first authorship.

## Abstract

**Introduction:**

The burden of influenza in Cambodia is not well known, but it would be useful for understanding the impact of seasonal epidemics and pandemics and to design appropriate policies for influenza prevention and control. The severe acute respiratory infection (SARI) surveillance system in Cambodia was used to estimate the national burden of SARI hospitalizations in Cambodia.

**Methods:**

We estimated age-specific influenza-associated SARI hospitalization rates in three sentinel sites in Svay Rieng, Siem Reap and Kampong Cham provinces. We used influenza-associated SARI surveillance data for one year to estimate the numerator and hospital admission surveys to estimate the population denominator for each site. A national influenza-associated SARI hospitalization rate was calculated using the pooled influenza-associated SARI hospitalizations for all sites as a numerator and the pooled catchment population of all sites as denominator. National influenza-associated SARI case counts were estimated by applying hospitalization rates to the national population.

**Results:**

The national annual rates of influenza-associated hospitalizations per 100 000 population was highest for the two youngest age groups at 323 for < 1 year and 196 for 1–4 years. We estimated 7547 influenza-associated hospitalizations for Cambodia with almost half of these represented by children younger than 5 years.

**Discussion:**

We present national estimates of influenza-associated SARI hospitalization rates for Cambodia based on sentinel surveillance data from three sites. The results of this study indicate that the highest burden of severe influenza infection is borne by the younger age groups. These findings can be used to guide future strategies to reduce influenza morbidity.

Influenza is a contagious, acute respiratory infection caused by influenza viruses. (*1*) Globally, seasonal influenza causes significant morbidity, mortality and socioeconomic costs. (*2*) Accurate figures of the burden of influenza are difficult to estimate. Robust vital statistics and civil registration, well functioning surveillance systems, hospital discharge databases and the expansion of influenza molecular testing have allowed more countries to complete influenza burden estimations. However, due to data quality and availability issues, the burden of seasonal influenza in low-income, lower middle-income and tropical climate countries is not well documented. Consequently, many countries lack influenza prevention and control policies. (*3*, *4*) Limited available data indicate that influenza burden in tropical settings, defined as areas with humid or arid/semiarid climates with mean temperatures of the coolest month above 18 °C, is higher than in temperate regions, particularly in children. (*5*) The prolonged circulation of seasonal influenza viruses in tropical areas could explain the higher burden. To address this data gap, the burden of influenza can be estimated using mathematical modelling. Recent estimates for the south-eastern Asian region indicate a considerable burden of influenza (> 100 000 deaths per year). (*6*)

Effective prevention and control strategies for influenza are assisted by routine seasonal influenza burden estimates based on local data. The earliest analysis of influenza-like illness (ILI) and severe acute respiratory infection (SARI) surveillance data available for Cambodia (2009–2011) indicated seasons with a predominance of A(H1N1)pdm09 and with co-circulation of influenza A(H1N1), A(H3) and influenza B. (*7*) Circulation of influenza A(H1N1)pdm09, influenza B and A(H3N2) was reported by ILI surveillance in 2010–2012 in Cambodia both in urban and rural areas. (*8*, *9*) In addition, the threat of avian influenza A(H5N1) in Cambodia (*10*) demands robust surveillance systems capable of monitoring the impact on hospitalization rates of novel influenza viruses associated with severe disease.

In 2006, the Virology Unit at the Institut Pasteur in Cambodia, the Communicable Disease Control Department of the Ministry of Health and the World Health Organization (WHO) country office jointly established a National Influenza Centre (NIC) in Cambodia. The aim of the NIC was to monitor and characterize circulating strains of influenza virus associated with mild and severe diseases. (*7*)

Since 2009, Cambodia has conducted hospital- and laboratory-based surveillance for SARI to characterize the epidemiology of severe respiratory illnesses associated with influenza A and B viruses and other common respiratory pathogens. (*11*) SARI surveillance in Cambodia is conducted throughout the year due to year-round influenza activity. (*7*) The objective of this study was to estimate the national influenza-associated hospitalization burden using SARI surveillance data.

## Methods

### Data sources

#### SARI sentinel surveillance sites

**Figure 1:**
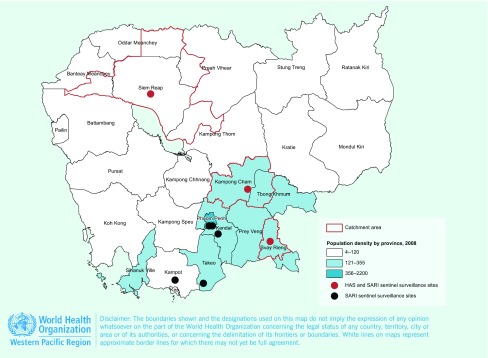
Map of Cambodia showing the eight SARI sentinel surveillance sites (black and red circles).*

SARI surveillance in Cambodia includes eight sentinel surveillance sites. For this study, sentinel sites were public health care inpatient facilities (HCFs) where SARI patients were identified and clinical, demographic information and respiratory specimens were collected. A SARI case was defined as measured fever (temperature ≥ 38 °C) or history of fever, and cough or sore throat, and shortness of breath or difficulty breathing in a hospitalized person with onset of symptoms within 10 days before hospitalization. (*12*) All data were recorded in a secure online database. Sentinel sites were located in Phnom Penh (two sites), Kandal, Siem Reap, Takeo, Kampong Cham, Svay Rieng and Kampot provinces (**Fig. 1**). New SARI cases were reported weekly by sentinel sites throughout the year. National virological and epidemiological surveillance data were reported in a monthly respiratory bulletin and published online. (*13*)

[**insert** Figure 1]

To estimate SARI rates, we used data from the three sentinel sites where Hospital Admission Surveys (HAS) had been conducted (**Fig. 1**). Two sites were rural and one was urban. Only three of the eight sites were included in the HAS due to resource limitations. Criteria used for site selection were site acceptance to participate in HAS and either the perceived quality of their data or availability of medical records in English.

Additional details on sentinel sites, case definitions and laboratory methods are available in **Appendix I** and **II**.

#### Hospital admission surveys

Hospital admission surveys were conducted in three locations to estimate the catchment population of each sentinel site using methods recommended by WHO (*14*) and piloted at the Svay Rieng sentinel site. (*15*, *16*) First, the addresses of the SARI cases admitted to the sentinel site were reviewed, and the catchment area for each site was defined as the districts from which 80% of the SARI cases admitted to the sentinel hospitals came (**Fig. 1**). We refer to the catchment area of each site as Svay Rieng, Siem Reap and Kampong Cham.

Second, we listed the non-sentinel health facilities in the catchment areas of the sentinel sites that admitted patients overnight. We visited these health facilities to enumerate respiratory admissions consistent with the following diagnoses: acute pulmonary oedema, asthma, asthma-pneumonia, bronchiolitis, bronchitis, broncho-asthma, broncho-pneumonia, flu/cold, laryngitis, lung abscess/empyema, pharyngitis, pneumonia, pneumopathy, pulmonary tuberculosis, respiratory infection, rhino-pharyngitis, severe pneumonia and tonsillitis. These diagnoses, which were collected from hospital log books, represent a proxy measure for SARI diagnosis. We collected information from 38 privately operated non-sentinel HCFs from 1 January–31 December 2015 (Svay Rieng site) and 1 January–31 December 2016 (Siem Reap and Kampong Cham sites). The data collection team (approximately 12 enumerators and four supervisors) used paper-based forms to collect data from eight non-sentinel HCFs in Svay Rieng, 16 in Siem Reap and 14 in Kampong Cham. Non-sentinel HCFs kept records in Khmer, French, Vietnamese and English. Enumerators captured data recorded in Khmer or English. HAS data were entered in data collection forms and subsequently entered into Epi Info 7 in English. (*17*)

We calculated the age-specific proportion of SARI cases that sought care at each sentinel site out of all respiratory admissions across all HCFs in the catchment area. Admissions from patients that resided outside the catchment area were excluded from both the numerator and the denominator. We assume the proportion of catchment population of the sentinel site to the total population is the same as the proportion of SARI cases seeking care in sentinel sites to SARI cases or respiratory admissions in all HCFs. Therefore, this proportion was applied to the age-specific district population (Ministry of Health Management Information System data) to generate an estimated catchment population for each sentinel site to be used as a population denominator for hospitalization rate calculations.

### Data validation

We compared the number of SARI cases reported through the surveillance system with the number of cases identified through manual review of paper-based medical records using the same case definition for six weeks both during and out of typical influenza virus circulation periods. In addition, we conducted staff surveys at two sites to explore acceptance and technical aspects of SARI surveillance (**Appendix III**).

### Data analysis

Site-specific annual hospitalization rates of influenza-associated SARI and 95% confidence intervals were calculated. For each site, we calculated the number of influenza-associated SARI hospitalizations by multiplying the age-specific influenza positive percentages in each month by the corresponding SARI case count in the same month. For sites with underreporting of SARI cases, we used SARI case counts identified by record review as a numerator in rate calculations by site.

To estimate national influenza-associated SARI hospitalization rates by age group, we used pooled data from the three sites. The count of SARI hospitalization nationally was calculated by multiplying the age-specific rates by the national population in the corresponding age groups. (*18*)

### Ethical approval

The hospital admission review consisted of a retrospective review of health data collected by the SARI sentinel surveillance system, which is a public health activity managed by the Cambodia Ministry of Health. The ethical aspects of this study were approved by the Australian National University Human Research Ethics Committee (Protocol 2017/337).

## Results

### Counting SARI cases at sentinel sites: findings from SARI surveillance

Overall, 2868 SARI cases were enrolled: 203 cases at Svay Rieng site, 922 cases at Siem Reap site and 1743 cases at Kampong Cham site. The majority of influenza-associated SARI cases in all sites combined were children under 5 years of age (51%) followed by the two older age groups (50–64 years and ≥ 65 years) representing 21% of SARI admissions (**Table 1**).

**Table 1:** Number of annual severe acute respiratory infection (SARI) cases and influenza-positive cases by age group and sentinel site, 1 January–31 December 2015 (Svay Rieng) and 1 January–31 December 2016 (Siem Reap and Kampong Cham, Cambodia)

Age group (years)	Svay Rieng			Siem Reap*			Kampong Cham			Total influenza- associated SARI cases
	SARI cases	Per cent positive for influenza^‡^	Influenza-associated SARI cases^†^	SARI cases	Per cent positive for influenza^‡^	Influenza-associated SARI cases^†^	SARI cases	Per cent positive for influenza^‡^	Influenza-associated SARI cases^†^	
< 1	8	0% (0/8)	0	455	10.4% (15/144)	47	381	10.0% (2/20)	38	85 (24%)
1–4	18	11.1% (2/18)	2	376	10.9% (19/175)	41	256	20.8% (10/48)	53	96 (27%)
5–15	6	33.3% (2/6)	2	91	10.0% (1/10)	9	157	30.0% (3/10)	47	58 (16%)
16–24	4	25.0% (1/4)	1	NA			91	12.0% (3/25)	11	12 (3%)
25–49	40	7.5% (3/40)	3				244	11.4% (8/70)	28	31 (9%)
50–64	61	6.6% (4/61)	4				280	10.0% (3/30)	28	32 (9%)
≥ 65	66	7.6% (5/66)	5				334	11.1% (5/45)	37	42 (12%)
Total	203	8.4% (17/203)	17	922	10.6% (35/329)	97	1743	13.7% (34/248)	242	356 (100%)

* Siem Reap sentinel site was a paediatric hospital and admitted children < 16 years of age.

‡ Per cent positive for influenza is the proportion of SARI cases that tested positive for influenza.

† Influenza-associated SARI cases were calculated by applying the age-specific influenza per cent positive for each month to the corresponding SARI case count for each month.

### Validation of SARI data at three sentinel sites

In Siem Reap, 259 records from patients hospitalized during six weeks in 2016 were reviewed and 98 met the SARI case definition. The surveillance system identified 55 of these cases, indicating that 56% of SARI cases were identified and enrolled in surveillance. In Kampong Cham, we reviewed 99 records from patients hospitalized during six weeks in 2016. Of these, 28 patients met the SARI case definition and only 19 of these were captured by the surveillance system (32% underreporting). In Svay Rieng, we did not find underreporting. Instead we found overreporting by the surveillance system (i.e. 50 SARI cases were reported by the surveillance system compared to 41 identified by medical records review). (*15*)

Some respondents of the staff surveys reported that surveillance activities represented an acceptable workload. Challenges identified in the survey included difficulties in obtaining consent for specimen collection in children, swabbing distressed children, difficulties in applying the SARI case definition due to incomplete or unclear medical histories, parental misunderstanding regarding the purpose of specimen collection, difficulties in applying the case definition to neonates and fear of reprimand if unable to collect specimens due to lack of parental consent. Through staff surveys we found that SARI surveillance underestimated SARI in infants and children as those without swabs were not counted as SARI.

**Figure 2:**
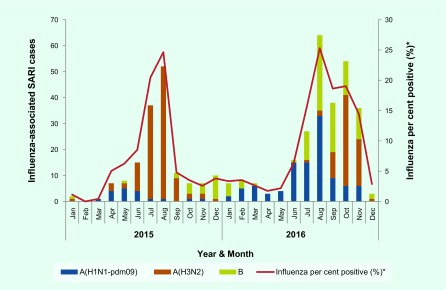
**Number of influenza-positive SARI cases by month and subtype/linage reported by all (eight) SARI surveillance sites, 1 January 2015–31 December 2016, Cambodia**

Influenza viruses circulated year-round with peaks in July and August. Multiple influenza virus types and subtypes were detected in 2015 and 2016; the predominant viruses were influenza A(H3N2) in 2015 and both A(H1N1)pdm09 and B in 2016 (**Fig. 2**).

[**insert** Figure 2]

### Estimated annual influenza-associated SARI hospitalization rate

The site-specific influenza-associated SARI hospitalizations rate varied widely. In 2015, the all-age influenza-associated SARI hospitalization rate in Svay Rieng was 7/100 000 population (**Table 2**). In 2016, the all-age rates in Kampong Cham were 72/100 000 population and much higher in the paediatric population (160). The combined influenza-associated SARI hospitalization rate was highest for children < 1 year (323/100 000 population) and 1–4 years (196) followed by those aged ≥ 65 years (91). Influenza-associated SARI hospitalization rates varied by site – with the largest differences seen in the < 1 years age group – from 0 for Svay Rieng to 495 per 100 000 in Kampong Cham. Hospitalization rates for Kampong Cham were higher compared with other sites for all age groups. Estimated age-adjusted influenza-associated SARI hospitalizations in Cambodia in 2016 were 7547 with most hospitalizations among children < 16 years of age (5328/7547).

**Table 2:** Estimated annual influenza-associated severe acute respiratory infection (SARI) hospitalization rate (and 95% confidence interval) by age group for each sentinel site and nationally, 2015 (Svay Rieng) and 2016 (Siem Reap and Kampong Cham, Cambodia)

Age group (Years)	Site-specific influenza-associated SARI hospitalization rate (HR) per 100 000 population*			Combined influenza-associated SARI HR per 100 000 population^†^ A	Cambodian population B	National influenza-associated SARI case count^‡^
	Svay Rieng§	Siem Reap^¦^	Kampong Cham			
< 1	0	345.4 (259.8–459.2)	494.9 (360.3–679.9)	323.0 (261.3–399.3)	348 518	1126 (1 060–2078)
1–4	14.8 (3.7–59.3)	206.1 (151.6–280.0)	338.2 (258.6–442.3)	196.0 (160.5–239.4)	1 235 655	2422 (2 325–4558)
5–15	4.7 (1.2–18.7)	33.2 (17.4–63.6)	195.7 (147.1–260.4)	61.8 (47.8–79.9)	2 880 177	1780 (1 697–3327)
16–24	1.9 (0.3–13.4)	NA	16.6 (9.2–30.1)	9.2 (5.1–16.6)	3 334 307	307 (272–534)
25–49	3.6 (1.2–11.2)		22.2 (15.3–32.2)	14.8 (10.4–21.1)	5 066 335	751 (697–1 366)
50–64	12.9 (4.8–34.4)		44.9 (31.0–65.1)	35.4 (25.1–49.7)	1 544 946	546 (501–981)
≥ 65	36.2 (15.0–86.9)		110.3 (79.9–152.1)	90.8 (67.4–122.4)	677 422	615 (566–1110)
Total	7.0 (4.4–11.3)	159.7 (131.0–194.9)	72.4 (63.8–82.1)	56.1 (50.6–62.2)	15 087 360	7547 (7 376–14 458)**

* Site-specific HRs were estimated using methodology described in the WHO Manual for Estimating Disease Burden Associated with Seasonal Influenza. (*14*) We divided A (Table 2 in Appendix IV) by D (Table 2 in Appendix IV) and multiplied by 100 000.

† The combined influenza-associated SARI hospitalization rate was estimated by adding influenza-associated SARI cases from all three HAS sites (i.e. adding column A for each site in Table 2 in Appendix IV), dividing by the sum of the three catchment populations (i.e. adding column D for each site in Table 2 in Appendix IV) and multiplying by 100 000.

‡ The national influenza-associated SARI case count was estimated by applying the combined influenza-associated SARI hospitalization rate (A) to the Cambodian population (B) and dividing by 100 000.

§ The influenza-associated SARI hospitalization rate for Svay Rieng was calculated using 2015 surveillance data (whereas data for Siem Reap and Kampong Cham used 2016 data). The Svay Rieng HR slightly differs from previously published rates (*15*) due to a different population data source used for their calculation.

¦ The sentinel site in Siem Reap was a paediatric hospital that admitted children under 16 years of age.

** The total national influenza-associated SARI case count was calculated as the sum of all values in the column.

Only two decimal places are displayed, but calculations used > 10 decimal places.

## Discussion

We present the first national burden estimate of severe influenza in Cambodia using hospital-based influenza surveillance data representing a climatically and demographically representative sample of hospitalizations in Cambodia in both rural and urban areas. Our findings indicate that influenza is an important contributor to hospitalizations in Cambodia particularly among children < 5 years of age. In two sites, we observed that infants (< 1 year) had the highest influenza-associated SARI hospitalization rates (345 and 495 hospitalizations per 100 000 population) followed by children aged 1–4 years (206 and 338 cases per 100 000 population). Our combined estimates of influenza-associated SARI hospitalizations in children are consistent with findings from African countries (*19*, *20*) but higher than those reported for Indonesia and India (82–114 and 118/100 000 children 0–4 years, respectively). (*21*, *22*)

When age-specific influenza-associated SARI hospitalization rates could be estimated across all age groups, we observed higher rates in infants and young children, lower rates in working-age adults and higher rates among those > 65 years of age. The same patterns of influenza burden have been reported in tropical climate countries. For example, the Lao People's Democratic Republic reported hospitalization rates of 156, 44, 9 and 42 per 100 000 population in 0–4, 5–14, 15–64 and 65 years age groups, respectively. (*23*) In both Zambia and Rwanda influenza-associated hospitalization rates in infants were highest compared to all other age groups (484 and 295/100 000 children < 1 year, respectively), and rates were lowest for the 5–24 years age group (6 and 11/100.000 5–24 years, respectively). (*19*, *20*) Compared to the hospitalization rates we estimated for older Cambodian adults, those reported for Zambia and Rwanda were lower (57 and 34/100 000 population > 65 years). (*19*, *20*)

The combined burden of influenza hospitalizations across all age-groups estimated for Cambodia (56/100 000 population) is similar to that reported for Zambia (44) (*19*) but higher than Rwanda (35) (*20*) and Indonesia (19). (*21*) Influenza hospitalization burden likely varies both within and between countries. This may be explained by virological, geographical, sociological (health care-seeking behaviour), underlying health status of the population and burden estimation approaches.

Consistent with previous reports from Cambodia, countries in the region and globally, (*7*, *21*, *24*) influenza activity was detected throughout the year with peaks between March and December. In 2015 the predominant strain was influenza A(H3N2), whereas in 2016 A(H1N1)pdm09 and B co-circulated. Influenza A(H3N2) typically causes more severe disease in children and older adults compared with other seasonal influenza strains. (*1*) Therefore, differences in the predominant strain may not entirely explain the lower rates observed in 2015 in Svay Rieng.

Several limitations were identified in this study. The burden of influenza for Svay Rieng was estimated using data from 2015, the first year of operation of surveillance, whereas the other sites used 2016 data, the second year of surveillance. Using data from well established systems collected in the same calendar year would improve comparability among sites and years. This is particularly important given that the predominant influenza circulating strains usually differ between years, which is associated with specific disease severity and therefore differing impacts on hospitalization rates. Additionally, multiple years of surveillance data are needed to reliably quantify the burden of influenza.

Furthermore, we estimated the burden of influenza based on three of the eight sentinel sites. The associated catchment populations for the sites included represent approximately 4% of the Cambodian population. This presents challenges to the representativeness of our estimates at the national level. We recommend further burden estimations using data from all sentinel sites captured in multiple calendar years, which was not possible in this study. Despite these limitations, our work indicates that the burden of severe influenza in Cambodia, particularly in children and the elderly, deserves consideration as it causes many thousands of hospitalizations annually. The economic costs associated with these hospitalizations, although not estimated in this study, would be substantial and could potentially be mitigated through interventions to reduce the influenza burden.

Through staff surveys at two sentinel sites we found that the surveillance system underestimated SARI in children at an unknown frequency (see **Appendix III**). In some cases parents refused specimen collection for their child. In addition, staff reported that swabbing infants was difficult and sometimes avoided. This would have resulted in a biased estimation of hospitalization rates for children.

We were unable to make a direct comparison between the rate of hospitalizations due to influenza and that of other diseases because of unavailability of complete national morbidity statistics in Cambodia. Challenges in the implementation of International Statistical Classification of Diseases and Related Health Problems, 10th Revision (ICD-10) have been documented. (*25*) Training physicians in writing diagnoses and strengthening the implementation of ICD-10 would allow future burden of disease studies to be improved by allowing contextualization with other diseases. Nevertheless, the percentage of those hospitalized with severe respiratory illness attributed to influenza in Cambodia (10.9% of all SARI hospitalizations, all-ages average) is comparable to that reported for Thailand (10.4%) and Indonesia (14%). (*21*, *26*)

One important strength of the study is the data validation conducted to understand the extent of underreporting and the potential surveillance operational challenges.

The results of this study can be used by the Ministry of Health in Cambodia to consider the introduction of influenza vaccination to reduce the impact of influenza-associated hospitalizations in the most vulnerable population groups: children and elderly people. Furthermore, this work underscores the value of investing in routine influenza surveillance in low–middle-income countries as key drivers of population health and pandemic preparedness.
